# Optimized combination of MALDI MSI and immunofluorescence for neuroimaging of lipids within cellular microenvironments

**DOI:** 10.3389/fchem.2024.1334209

**Published:** 2024-02-09

**Authors:** Catelynn C. Shafer, Elizabeth K. Neumann

**Affiliations:** Department of Chemistry, University of California Davis, Davis, CA, United States

**Keywords:** multimodal, mass spec imaging, immunofluorecence, neuroscience, astrocyte, mass spectrometry

## Abstract

Proper neurological function relies on the cellular and molecular microenvironment of the brain, with perturbations of this environment leading to neurological disorders. However, studying the microenvironments of neurological tissue has proven difficult because of its inherent complexity. Both the cell type and metabolomic underpinnings of the cell have crucial functional roles, thus making multimodal characterization methods key to acquiring a holistic view of the brain’s microenvironment. This study investigates methods for combining matrix-assisted laser desorption/ionization mass spectrometry imaging (MALDI MSI) and immunofluorescence (IF) microscopy to enable the concurrent investigation of cell types and lipid profiles on the same sample. In brief, 1,5-diaminonaphthalene (DAN), α-cyano-4-hydroxy-cinnamic acid (CHCA), and 2,5-dihydroxybenzoic acid (DHB) were tested in addition to instrument-specific parameters for compatibility with IF. Alternatively, the effects of IF protocols on MALDI MSI were also tested, showing significant signal loss with all tested permutations. Ultimately, the use of CHCA for MALDI MSI resulted in the best IF images, while the use of DAN gave the lowest quality IF images. Overall, increasing the laser power and number of shots per laser burst resulted in the most tissue ablation. However, optimized parameter settings allowed for minimal tissue ablation while maintaining sufficient MALDI MSI signal.

## Introduction

The inherent complexity of biological systems is crucial for proper biological function (Nowinski and Miller, 2011). *The brain is fundamental for many critical biological functions, such as proper motor function, memory, and reasoning*. As a whole, the brain is comprised of approximately 100 billion cells, including neurons, astrocytes, endothelial cells, oligodendrocytes, and microglia with numerous cell subtypes (Nowinski and Miller, 2011; Herbet and Duffau, 2020). These cell types in the brain work together for proper motor function, cognition, and memory; therefore, cognitive functionality heavily relies on maintaining the elaborate cellular and molecular environment in the brain (Rutkowsky et al., 2018; Moujalled et al., 2021; Alomari et al., 2022; Mietelska-Porowska et al., 2022). Many neurological diseases such as Alzheimer’s disease, frontotemporal dementia, and amyotrophic lateral sclerosis can be attributed to changes in the single-cell metabolism, which result in an altered metabolomic profile, thus affecting the function of cells (Rutkowsky et al., 2018; Alomari et al., 2022; Mietelska-Porowska et al., 2022). The complex interplay between cellular and molecular components in the brain poses challenges to comprehensive and detailed characterization. Therefore, an additional methodology is needed for the proper understanding of and developing therapeutics for neurological disease.

**FIGURE 1:**
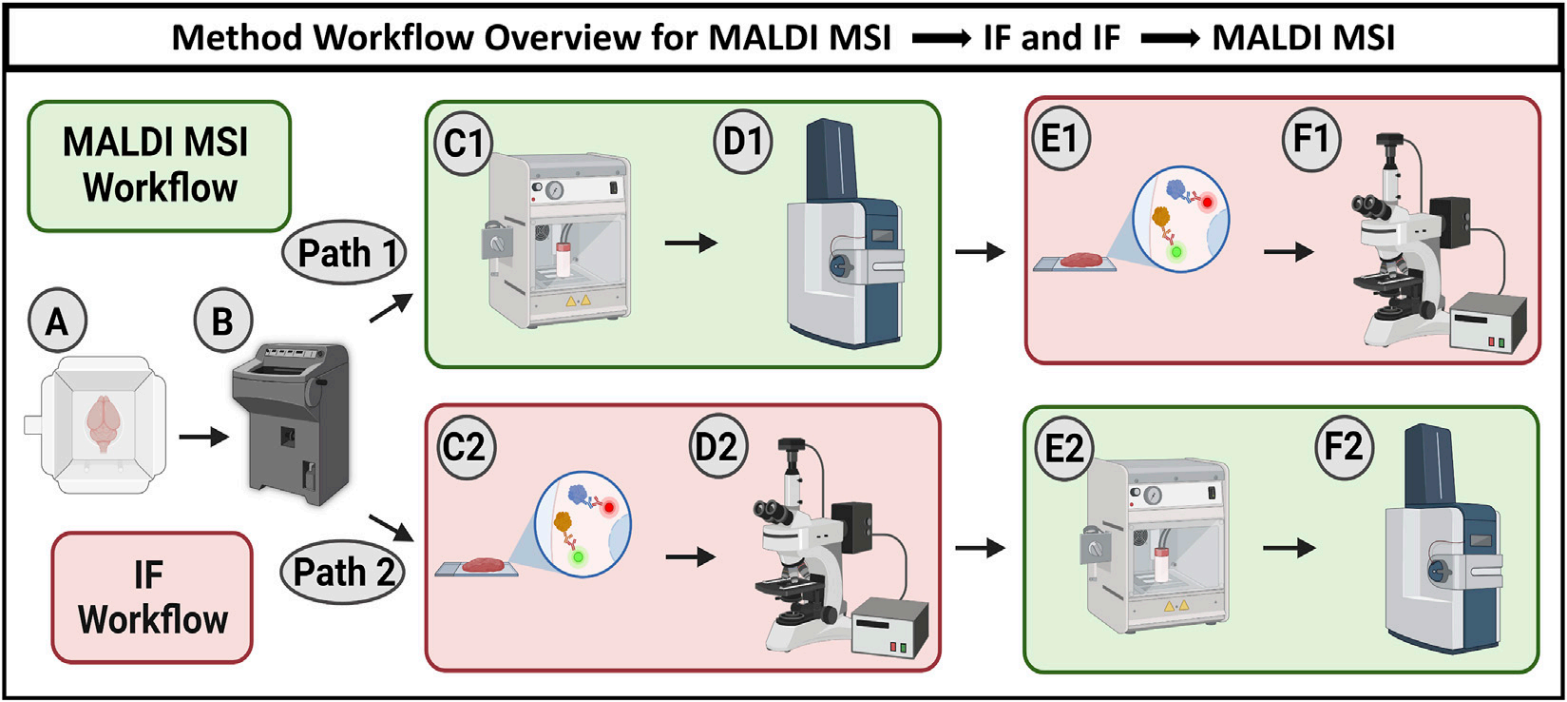
Workflow schematic showing two options for combining MALDI MSI and IF microscopy. The workflow begins with cryosectioning fresh frozen samples to 10 µm thickness **(A, B)**. Path 1 describes MALDI MSI being performed prior to IF microscopy. Tissue is sprayed with MALDI matrix (C1) and analyzed using MALDI MSI (D1) before antibody staining (E1) and subsequent fluorescence microscopy (F1). Path 2 describes the sequence where IF microscopy is performed prior to MALDI MSI. The tissue was first stained with antibodies (C2) and imaged with fluorescence microscopy (D2) before being sprayed with MALDI matrix (E2) and analyzed using MALDI MSI (F2).

There are several existing methods for characterizing components of biological tissue, including immunochemistry (cell type/proteome), mass spectrometry (metabolome/proteome), or transcriptomics (gene expression) (Burgess, 2019; Im et al., 2019; Kulkarni et al., 2019; Magaki et al., 2019; Bonacho et al., 2020; Tan et al., 2020; Alseekh et al., 2021; Rozanova et al., 2021). Obtaining experimental data from a single tissue sample is required to understand the links between metabolic molecules and cell or tissue types, making it advantageous to develop procedures that combine multiple characterization methods. Some existing methods for cellular identification include hematoxylin and eosin (H&E) staining, immunofluorescent (IF) antibody staining, RNA sequencing, and Golgi staining (Zhong et al., 2019; Hong et al., 2020; Rodig, 2022; García-García et al., 2023). Metabolomics analysis can be accomplished via liquid chromatography–mass spectrometry (LC-MS); however, collecting metabolomic information of cellular tissue while maintaining spatial distribution requires different methods such as matrix-assisted laser desorption/ionization mass spectrometry imaging (MALDI MSI), secondary ion mass spectrometry (SIMS), or desorption electrospray ionization mass spectrometry imaging (DESI MSI) (Rutkowsky et al., 2018; Kaya et al., 2020; He et al., 2022). Here, we use MALDI MSI, owing to its broad chemical coverage and availability, although similar experiments could be designed for DESI or SIMS as well. MALDI MSI uses a UV laser assisted by an organic matrix to ionize and desorb endogenous molecules from the tissue surface. The MALDI source is positioned on a variable stage, allowing the laser to raster through an assigned grid, spatially mapping hundreds of thousands of mass spectra. This process enables visualization of the relative abundance and spatial distribution of each detected molecule. MALDI MSI has been used for metabolomic imaging in samples such as kidney, brain, and liver (Djambazova et al., 2020; Kaya et al., 2020; Zhou et al., 2021; He et al., 2022). Immunofluorescence (IF) is the gold standard for labeling cell types/states. In general, IF uses a primary antibody that is directly or indirectly connected to a fluorophore to visualize the distribution of the targeted protein associated with a known cell type, allowing for cell type/state analysis (Im et al., 2019; Francisco-Cruz et al., 2020; Zaqout et al., 2020; Elsborg et al., 2023). This study focuses on developing methods for combining MALDI MSI and IF, demonstrating how parameter and procedural variations affect MALDI MSI or IF results within wild-type murine brain samples. Using the same tissue sample for both MALDI MSI and IF allows for true cell-to-cell comparisons that are not possible with serial sections. However, due to the significant difference in sample preparation between MALDI MSI and IF experiments, it is challenging to perform both methodologies on the same sample. Multimodal approaches that combine MALDI MSI and IF have been used in heart, brain, and esophagus, allowing for rich analysis of the molecular and cellular environment of the tissue, although some challenges in this multimodal approach still remain, including mismatched spatial resolution or procedure compatibility (Huber et al., 2019; Jones et al., 2019; Kaya et al., 2020; Kaya et al., 2021). Nonetheless, recent work demonstrates that it is possible to perform MALDI MSI and IF on the same tissue section (Dunne et al., 2023; Oyler et al., 2023). Here, we assess the effects of common variables, such as MALDI matrix choice, MALDI instrumental settings, fixation length, and detergent choice, on MALDI MSI and IF when performed on the same tissue. Exploiting the strengths of both methods yields a richer, more comprehensive dataset describing the molecular and cellular microenvironment of tissue. There are two possible workflows for the multimodal application of MALDI MSI and IF microscopy (Figure 1). Both methods are compatible with fresh frozen, cryosectioned tissue, regardless of whether the tissue is embedded or not. After thaw mounting, the experimental processes between MALDI MSI and fluorescence microscopy diverge due to no overlap in the preparation steps between the two methods. As such, one entire process must be completed before preparations for the second can begin. MALDI MSI can be performed prior to IF microscopy (Figure 1, path 1) or *vice versa* (Figure 1, path 2). Both workflows require identical time and materials; therefore, the superior workflow must be determined by the quality of the resulting data, considering that both the MALDI MSI and IF microscopy contain critical insights into biological systems, and these are explored herein.

## Methods

### Tissue preparation

For all experimental methods, chemicals were purchased from Thermo Fisher Scientific unless otherwise specified. A total of three fresh frozen wild-type mouse brains were used. Prior to sectioning, the samples were taken from −80°C and allowed to equilibrate to −20°C for 30 min inside a cryostat (Leica Biosystems, Wetzlar, Germany). Sagittal sections of the mice brains were cut to 10 µm and thaw mounted onto indium tin oxide (ITO) slides (Delta Technologies, Limited, Loveland, Colorado) and stored at −80°C until further analyzed. For matrix comparison and immunofluorescence experiments, a total of three brains were used, with serial sections being used for each tested matrix for a total of nine sections. For method parameter experiments, four serial sections from the same brain were used. This was done to minimize error in the resulting MSI data caused by cellular differences between sections and to make the methods as comparable as possible.

### Matrix application

For matrix application, slides were removed from a −80°C freezer and returned to ∼20°C in a vacuum desiccator for 30 min or immediately after IF experiments were performed. Moreover, 1,5-diaminonaphthalene (DAN, Tokyo Chemical Industry co, Tokyo, Japan), α-cyano-4-hydroxy-cinnamic acid (CHCA, Sigma-Aldrich, St. Louis, MO), and 2,5-dihydroxybenzoic acid (DHB, Sigma-Aldrich, St. Louis, MO) were all used for the MALDI MSI experiments without further purification. The matrix solutions included 20 mg/mL DAN in tetrahydrofuran, 5 mg/mL CHCA in 70% methanol, and 40 mg/mL DHB in 50% methanol. An HTX TM M3 Sprayer (HTX technologies, LLC, Chapel Hill, NC) was used for matrix application. DAN was applied with a flow rate of 0.05 mL/min, 1,350 mm/min, 5 passes, 40°C nozzle, and no drying time. CHCA was applied using a flow rate of 0.120 mL/min, 1,200 mm/min, 8 passes, 80°C nozzle, and a 4-s drying time. DHB was applied using a flow rate of 0.05 mL/min, 1,250 mm/min, 24 passes, 80°C nozzle, and no drying time. MALDI MSI was performed on the same day as the matrix application. The resulting matrix density for CHCA, DAN, and DHB was 0.12 mg/cm^2^, 0.09 mg/cm^2^, and 1.31 mg/cm^2^, respectively. When MALDI MSI was performed after IF, the tissues were rinsed three times for 5 min each time in PBS before being coated with CHCA according to the previously described procedure. The MALDI method used was the same for the matrix comparison experiments.

### Matrix comparison experiments

All MSI experiments were performed using a Bruker MALDI timsTOF FleX mass spectrometry system (Bruker Scientific, Billerica, MA). All three slides were initially run using the same method, and this resulted in an adequate signal for CHCA- and DAN-sprayed tissues but no signal for DHB-sprayed tissues (SI Supplementary Figure S1). Because of this, a greater laser power of 67% was required for the DHB-coated samples. Select positive mode settings included 150 shots, 10 kHz laser frequency, 40% laser power, and either 50 or 30 µm spatial resolutions. The global laser attenuator was set to 0%. SCiLS lab (Bruker Scientific, Billerica, MA) was used to process and visualize the data.

### MALDI MSI parameter experiments

The method described in “Matrix Comparison Experiments” was the base method, and one MALDI-related variable was changed at a time from this base method. These variables included the number of laser shots (50, 100, 500, and 1,000), laser frequency (1kHz, 2kHz, 5kHz, and 10 kHz), and laser power (30%, 50%, 75%, and 100%). Four tissues from one brain were used for the method parameter experiments. Of these tissues, three were acquired with a 100 µm spatial resolution and one was acquired with a 30 µm spatial resolution to assess the affects at higher spatial resolutions.

### Immunofluorescence microscopy

Samples used for immunofluorescence were thawed in a desiccator at ∼20°C for 30 min unless MALDI MSI was performed previously. Thawed tissues were fixed in a neutral buffered formalin for 15 min. Slides that had previously undergone MALDI MSI were fixed for the same amount of time; however, the formalin was replaced after the first 5 min because the removed matrix changes buffer acidity and may affect fixation efficacy. The samples were rinsed with phosphate-buffered saline (PBS, G-Biosciences, St. Louis, MO) three times for 3 min each time, blocked for 45 min with 50 mg/mL bovine serum albumin (BSA, Roche Diagnostics GmbH, Mannheim, Germany) and 0.5% Tween 20 (Sigma-Aldrich, St. Louis, MO) or triton 100x (Sigma-Aldrich, St. Louis, MO) in PBS, and rinsed with PBS three times for 3 min each time. A hydrophobic pen (Daido Sangyo Co, Tokyo, Japan) was traced around the tissue area for minimizing the amount of antibody solution that was applied. For direct fluorescence experiments, tissues were incubated overnight at 4°C in antibody solution (1% BSA and 0.5% Tween 20 in PBS) with a 1/500 dilution of anti-GFAP Alexa fluor 488 (Invitrogen, Clone GA5, Lot 2497614) primary antibody dilution. Hoechst 33342 (ANASPEC INC, Fremont, CA) was added 30 min before rinsing with PBS three times for 5 min. For indirect fluorescence experiments, tissues were incubated overnight at 4°C in antibody solution with a 1/500 dilution of anti-GFAP antibodies grown in mouse (Clone CL2713, lot MAB-03334, Sigma-Aldrich, St. Louis, MO). Slides were rinsed in PBS three times for 5 min, the hydrophobic pen was reapplied, and the antibody solution was applied with a 1/100 dilution of Alexa fluor 594 donkey anti-mouse IgG (lot 2474956). Hoechst 33342 was added 30 min before rinsing. The tissues were rinsed in PBS three times for 5 min each time. For both direct and indirect, fluorescence mounting media (Millipore Corp, Burlington, MA) were applied, and the slides were cover slipped and imaged on a fully automated EVOS M7000 fluorescence microscope (Thermo Fisher Scientific Inc, Waltham, MA) using the 357/447 nm, 470/525 nm, and 531/593 nm channels and autofocused at every five frames. The high-resolution images were stitched and analyzed using Fiji open source software (Schindelin et al., 2012). Indirect antibodies were used for samples that had already undergone MALDI MSI experiments, including the matrix comparison experiments and the method parameter experiments.

Fixation time experiments were conducted using neutral buffered formalin for 15 min, 30 min, 1 h, 3 h, and 24 h on different tissues (three tissues for each timepoint). PBS washes were performed three times for 3 min each between steps. After fixation, all tissues were blocked with blocking buffer (50 mg/mL bovine serum albumin (BSA) and 0.5% Tween 20 in PBS) for 45 min and rinsed with PBS. They were then dried and immediately sprayed with CHCA. These samples were kept in a desiccator until run on the MALDI timsTOF flex using the same lipid method as the matrix comparison experiments. CHCA matrix was applied to the post IF samples after being rinsed three times with PBS for 5–10 min each time.

### Astrocyte analysis

Three groups of tissues were included in the astrocyte analysis: MALDI MSI performed before IF, direct IF performed before MALDI MSI, and indirect IF performed before MALDI MSI (n = 3 for each group). All tissues underwent both MALDI MSI and IF, although in different orders. The IF images were coregistered with the MALDI MS images using SCiLS lab (Bruker Scientific, Billerica, MA). The IF images were used to identify 50 astrocytes in each tissue from the granular layer of the cerebellum and enabled identification of the lipid profiles for each astrocyte. Only astrocytes that had a single mass spectrum were used, reducing the likelihood of contamination from neighboring cells.

## Results and discussion

### Effects of matrix selection on immunofluorescence

**FIGURE 2:**
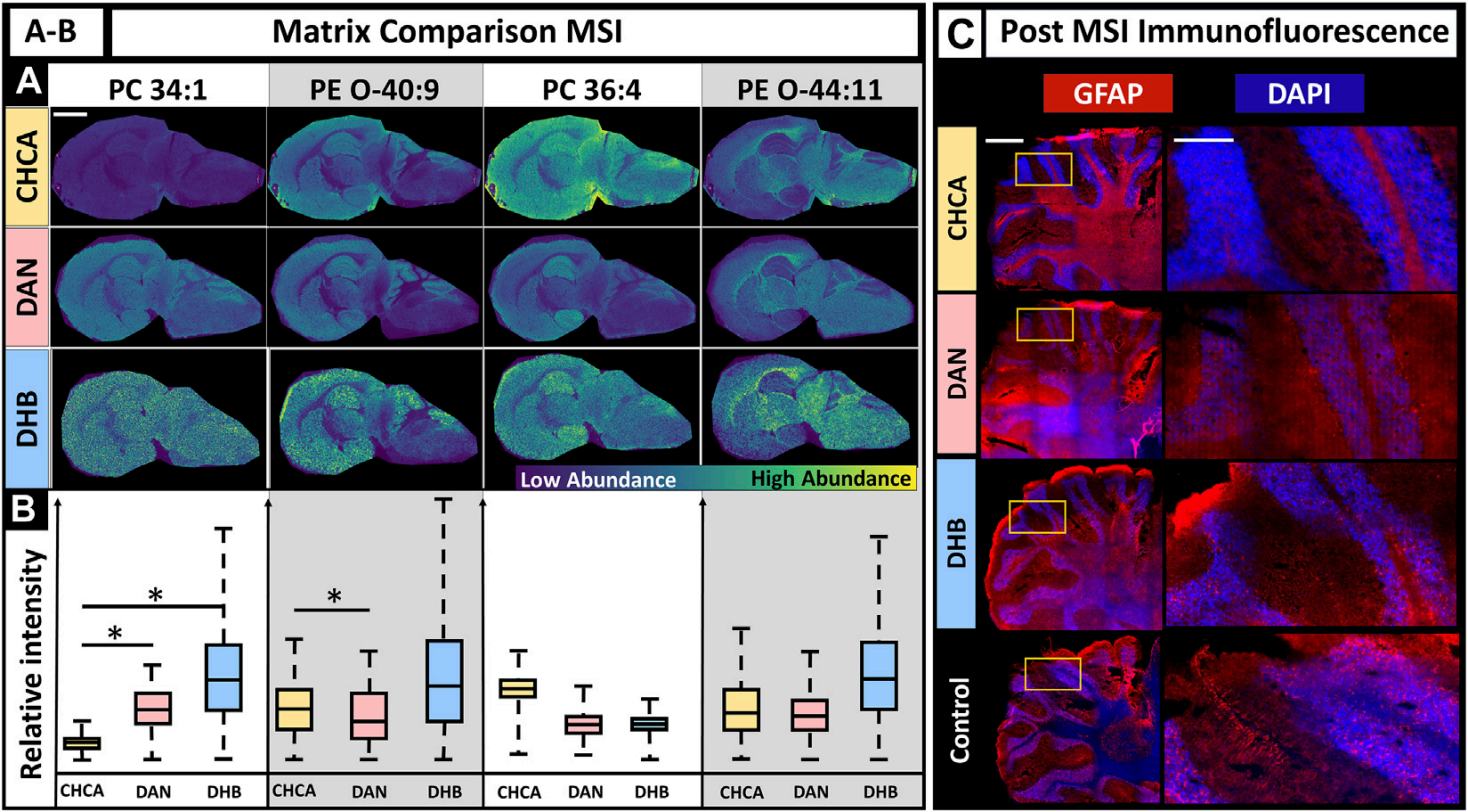
Effects of matrix choice on IF when MALDI MSI is performed first. **(A)**. MALDI MSI was performed on murine brains coated in CHCA, DAN, and DHB with the MS images of PC(34:1), PE (O-40:9), PC(36:4), and PE (O-44:10) as a demonstration (scale-bar 3 mm). **(B)**. Box plots show relative ion abundance of PC(34:1), PE (O-40:9), PC(36:4), and PE (O-44:10) for CHCA (yellow), DAN (red), and DHB (blue), respectively. The relative signal intensity was significantly different (two-tailed *t*-test) between CHCA and DAN for at least two lipids, PC(34:1) and PE (O-40:9). **(C)**. Fluorescence images after matrix application for CHCA, DAN, and DHB compared to control (left scale-bar 1 mm, right scalebar 0.25 mm). GFAP marker is red, and DAPI is blue. N = 3 for all conditions. * Indicates *p*-value <0.05.

The selection of an appropriate matrix is a crucial step for successful MALDI MSI experiments. The matrix enables ionization and desorption for specific molecular classes and method polarities. Factors to consider when choosing a matrix include the molecular class of interest (e.g., lipids, small molecules, proteins, glycans), ionization polarity (i.e., negative or positive ion mode), matrix volatility, MALDI laser wavelength, minimal background signal, and, in this case, the ability to gently remove the matrix for fluorescence experiments (Zhou et al., 2021). Three common matrix selections for MALDI MSI of lipids in positive mode are CHCA, DAN, and DHB, although DAN is capable of ionizing in positive and negative ion modes. The primary factors we considered were lipid coverage, MSI image quality, and fluorescence staining quality after the tissue had undergone MSI experiments. Due to matrix specific differences such as ionization efficiency, MALDI MSI parameters often vary depending on the matrix used. The method for this experiment used 150 shots to ensure sufficient ion signal and the lowest laser power that retained ion signal. A laser power of 40% was used for DAN, CHCA, and DHB, with the DHB samples repeated with a laser power of 67% due to insufficient ionization at 40% (SI Supplementary Figure S1). The MALDI MS images for DAN, CHCA, and DHB show that higher MS signal intensity does not necessarily indicate better-quality MS images, as demonstrated by DHB often having the highest signal intensity, but in addition to artifacts (Figure 2, PC 34:1). DAN and CHCA had similar signal localization in the MS images with lipid-dependent intensity variation. DHB had a higher signal intensity across a larger range of lipids; however, the resulting MALDI MSI images had higher background, which may have resulted from the higher laser energy that was needed, delocalization, or other matrix application-related factors. The relative signal intensity for each matrix was lipid dependent, although no broad statements could be made for lipid class. While some significant differences in relative signal intensity between DAN and CHCA were observed, neither matrix showed consistently higher signal intensity over the others (Figure 2C). Post-MSI tissues were washed and used for IF microscopy. CHCA and DHB had no visible residue on the slide after washing, but some DAN remained on the slide after following the same fixation procedure. The resulting fluorescence images show the effects of each matrix (Figure 2C). CHCA allowed for sufficient staining for nuclei and astrocytes via DAPI (blue) and glial fibrillary acidic protein (GFAP, red) immunofluorescent staining. DAN caused poor staining for both nuclei and astrocytes and was the only matrix to leave visible laser ablation marks on the tissue, despite the CHCA-coated sample having the same laser parameters and the DHB coated sample requiring higher laser energy. DHB, much like CHCA, allowed for sufficient staining for both nuclei and astrocytes. As a result, the use of CHCA under these conditions allowed for sufficient MSI signal intensity with the least number of artifacts compared to DHB or DAN matrix coatings and sufficient post-MSI immunofluorescence staining.

### Parameter experiments

**FIGURE 3:**
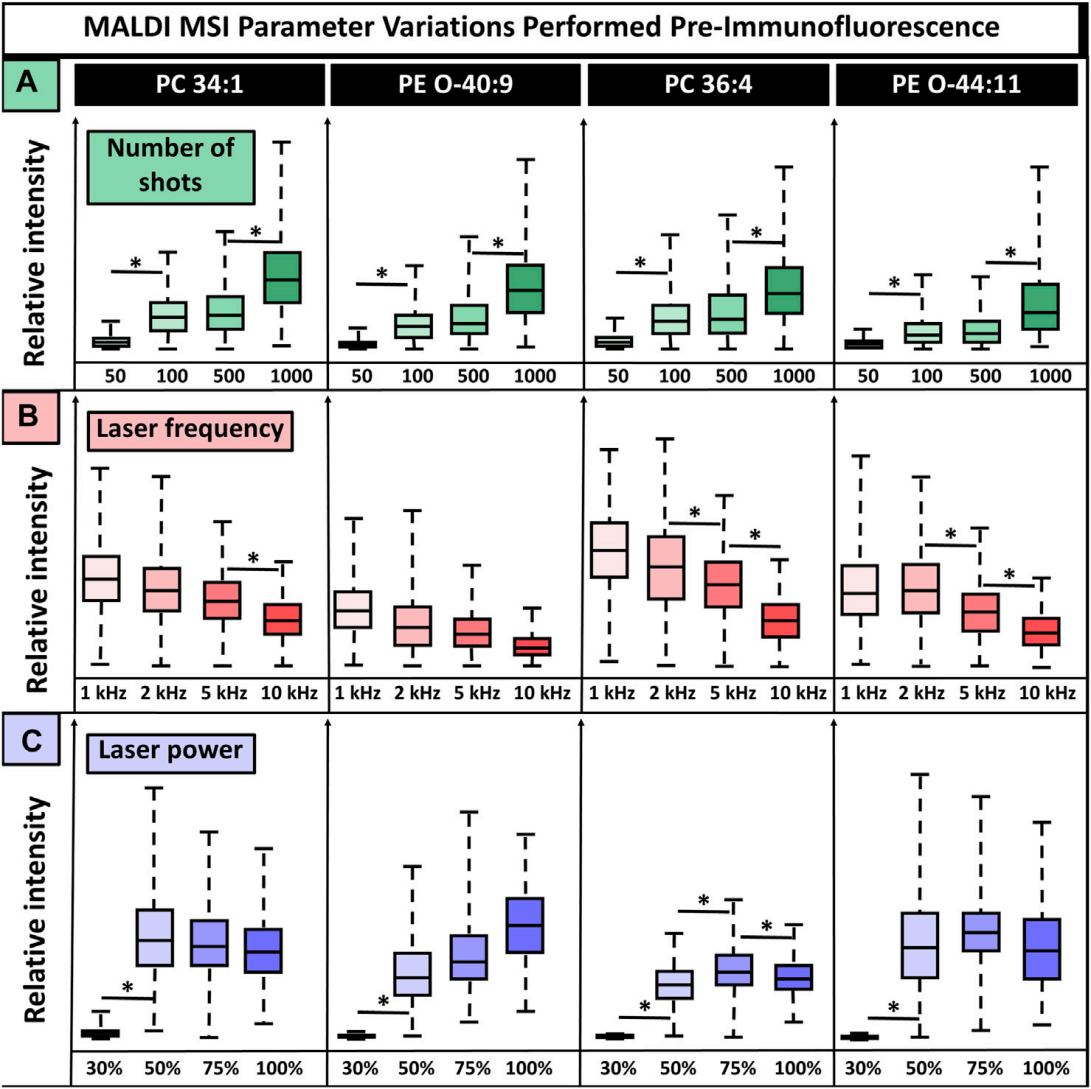
MALDI laser parameter settings without normalization show the relative ion signal intensity for four lipids. **(A)**. In total, 50, 100, 500, and 1,000 laser shots were tested, with increasing shots leading to increased signal intensity for all lipids. **(B)**. Frequencies of 1, 2, 5, and 10 kHz were tested, and an inverse effect on signal intensity was detected, in which increasing frequency resulted in lower signal intensity. **(C)**. Laser powers of 30%, 50%, 75%, and 100% resulted in lipid-dependent variation, and no consistent conclusion could be drawn. The number of shots, laser power, and laser frequency were tested on the cerebellum, midbrain, and break stem, respectively. Four serial sections were used for all conditions. * Indicates *p*-value <0.05 from a two-tailed *t*-test.

In addition to matrix selection, the MALDI MSI method parameters are critical. Laser settings affect the deterioration and destruction of the tissue and, in turn, the quality of subsequent IF images, making it a crucial feature requiring refinement. Laser power, laser frequency, and the number of laser shots affect the ionization of endogenous molecules and resultant signal intensity while simultaneously contributing most to tissue deterioration. Because of this, these three parameters were selected for testing the parameter settings that resulted in the highest signal intensities while not introducing artifacts in IF images (Figure 3). CHCA was used for method parameter experiments since it demonstrated the least tissue damage and interference in IF experiments while maintaining broad ion detection with minimal artifacts. We anticipate the following conclusions to many matrices, but these may need to be individually tested. Parameters were adjusted one at a time to observe their effects on signal intensity. We tested 50, 100, 500, and 1,000 laser shots, laser frequencies set to 1 kHz, 2 kHz, 5 kHz, and 10 kHz, and laser powers at 30%, 50%, 75%, and 100% power with the global attenuator set to 0% (the laser was <3 months old). Some parameters had lipid-dependent signal variations, while others showed consistent trends for all lipids. For instance, regardless of the lipid, there was increased relative ion abundance with an increased number of shots, whereas laser power had a lipid-dependent effect. Overall, 50 laser shots were insufficient for adequate lipid ionization and gave statistically significant (p-valued <0.05, two-tailed *t*-test) signal intensity differences that were eight times lower than observed when using >100 shots. Furthermore, 100 shots and 500 shots produced an equivalent signal intensity with no statistical variation, while 1,000 shots gave a two times higher signal intensity that was statistically significant (p-valued <0.05, two-tailed *t*-test), relative to 100 and 500 shots. The highest signal intensity was produced using 1,000 shots, but this resulted in some artifacts in both the MSI and IF (SI Supplementary Figure S2). This was likely a result of oversampling. Regardless of the *m/z* value, increased laser frequency caused decreased signal intensity. For three of the four lipids shown, there was a statistically significant drop in signal intensity, albeit ions were still detected at all tested frequencies. Then, 10 kHz was selected for subsequent experiments because of its speed relative to the other frequencies. The speed of MSI experiments is crucial for throughput and efficiency since instrument run time can range between 3 and 24+ hours for one sagittal mouse brain section, depending on frequency and spatial resolution. This throughput becomes more critical when large numbers of samples are required to understand complex and heterogeneous diseases or variables such as diet, meaning the benefits of using the higher frequency outweigh the loss in signal for some cases, although samples where high sensitivity is needed may benefit from using a lower laser frequency. Laser power had a lipid-dependent effect on relative ion intensity, with 30% laser power being insufficient for ionization, although this laser power will vary based on global attenuator settings and laser age. In approximately half of lipids, 100% laser power caused a decrease in relative ion intensity that was significant in at least one case, PC(36:4) (p-valued <0.05, two-tailed *t*-test). This could result from fragmentation, saturation of the detector, or other issues. Additionally, such high laser energy will certainly cause tissue damage, indicating 100% laser power is not ideal for multiplexed experiments utilizing 100+ laser shots per pixel, as was done in this study.

### IF post-MSI parameters

**FIGURE 4:**
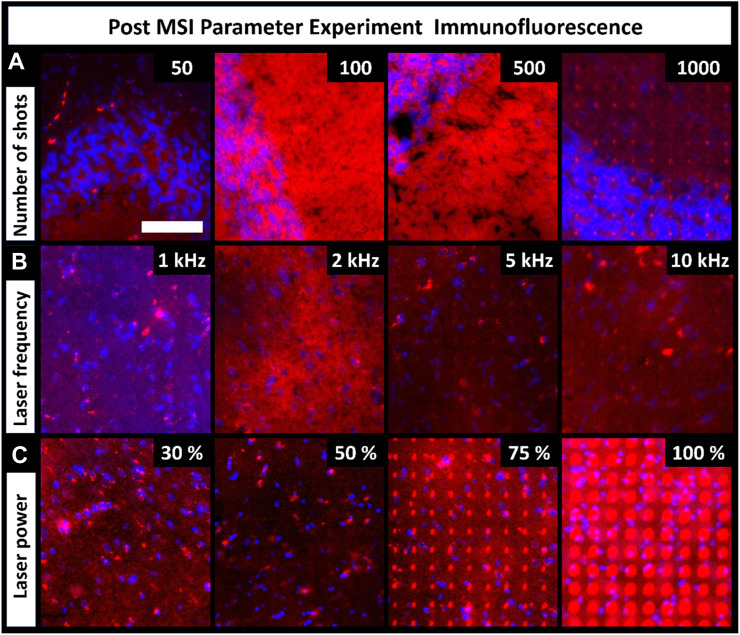
Fluorescence images of tissues previously used for MALDI MSI parameter experiments show the level of tissue damage caused by each parameter adjustment (scale-bar 100 µm). **(A)**. Tissue ablation increased with increasing number of shots. **(B)**. Laser frequency had no visible effects on tissue ablation. **(C)**. Laser power had an increasingly destructive effect on the tissue. Four serial sections were used for all conditions (these tissues were the same as the ones discussed in Figure 3).

MALDI uses laser energy to desorb and ionize molecules, making it destructive to the tissue surface. The level of tissue ablation depends on various laser parameters, such as shot count, frequency, and laser energy. These parameters are critical components of MALDI MSI, so we explored how they affect tissue damage/distortion in resultant immunofluorescence images. Tissue ablation varied between the parameters and their settings (Figure 4). Parameter experiments were performed on CHCA-coated tissue. Tissue ablation differs when another matrix is used, as observed in the artifact differences between the CHCA- and DAN-sprayed tissues (Figure 2). Increasing the number of laser shots should theoretically increase tissue ablation; however, the number of shots had no visible effects on tissue ablation until 1,000 shots were used (Figure 4A). Ultimately, the required number of shots varies based on factors such as instrument cleanliness and tissue type, but using more shots inevitably results in more tissue damage. Unlike the number of shots, changing laser frequency did not change tissue ablation, and minimal tissue ablation was observed in all regions (Figure 4B). Laser power was expected to have a direct effect on tissue ablation, which was seen to be the case (Figure 4C). Overall, 30% laser power resulted in the least tissue ablation; however, it had insufficient ion production in MSI spectra for broad chemical coverage. Additionally, 50% laser power showed minimal ablation, but 100% laser energy resulted in more severe tissue damage. Given the direct relationship between tissue damage and laser power, setting the laser power as low as possible to produce sufficient signal in MSI experiments preserved the sample from unnecessarily high levels of laser damage visible in IF images. As with the number of shots, the laser power required for optimized signal is influenced by external factors, such as instrument cleanliness, tissue type, or laser age and therefore varies.

### IF direct vs. indirect, IF post-MSI, fixation

**FIGURE 5 F5:**
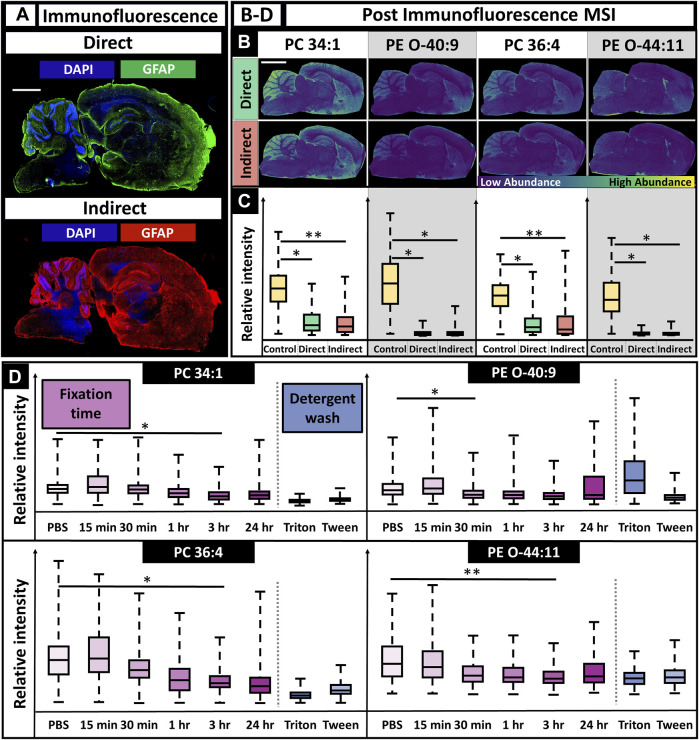
IF microscopy followed by MALDI MSI using directly and indirectly conjugated fluorescent antibodies and various durations of tissue fixation time. **(A)**. Immunofluorescence images for direct and indirect antibody conjugation showed adequate staining for astrocytes and nuclei (scalebar 1.5 mm; tissue edge had appreciable autofluorescence as is typical). **(B)**. MS images post-IF showed poor spatial mapping for all lipids (scalebar 3 mm). **(C)**. For all lipids, there was decreased MS signal when compared to controls (two-tailed *t*-test). **(D)**. Fixation times of 15 min, 30 min, 1 h, 3 h, and 24 h were all tested and had a lipid dependent effect on signal, although lipid signal generally decreased with increased fixation time **(D)**. N = 3 for all conditions. * Indicates *p*-value <0.05; ** Indicates *p*-value <0.10.

Given the MSI and IF workflow results, we determined MALDI MSI can be performed prior to IF and result in sufficient MS and fluorescence images when the described methods are utilized. However, this does not indicate that performing MSI prior to IF is the preferential workflow. To determine if MALDI could be performed after IF, the reverse workflow was completed. Both direct and indirect fluorescence experiments were tested and gave similar results, allowing for expanded flexibility (Figures 5A,B).

Performing IF experiments prior to MSI allowed for improved antibody staining for both nuclei and astrocytes as expected (Figure 5A). However, the MS images showed at least a statistically significant signal loss of 20% for all lipids, with losses up to 95% for some lipids. This made it difficult to determine specific features, such as the hippocampus, striatum, corpus collosum, and thalamus nuclei, that were previously discernible when only performing MSI. This is especially apparent for PE (O-44:10) (Figures 2A,5B, [Fig F5]). Molecular discrimination of brain regions are crucial in neuroscience studies since functionality is linked to specific regions, such as long-term memory, the hippocampus or gross motor function, and the frontal lobe (Pessoa, 2014). The severe loss in signal is multifaceted, resulting from a combination of the detergent-removing lipids and the fixative cross-linking metabolites, making them less available for desorption, with ionic interference from the solutions required for antibody staining, such as PBS. Delocalization effects of the lipids after the numerous rinses and fixation steps used in IF procedures are also a likely factor. As a result, performing MALDI MSI after fluorescence caused a consistent decrease in relative ion intensity when compared to a control sample, regardless of the *m/z* value (Figure 5C). Tissues fixed for varying amounts of time were analyzed using MSI to determine if there was an optimal fixation time that would allow for the retention of ion signal. Regardless of the *m/z* value, there was a decrease in relative ion intensity as the fixation time increased, with lipid-dependent effects that caused statistically significant signal loss after 1 h fixation for some lipids and 3 h for others (Figure 5D). Detergent had have little effect on the outcome of signal intensity.

### Comparison of the mass spectra

The mass spectra were generated by averaging all pixels (individual spectra) from the whole tissue, giving an overview of the lipidomic profile of the sample. The mass spectra resulting from pre-immunofluorescence staining (Figure 2) and post-IF staining (Figure 5) show that matrix selection and tissue treatment prior to matrix application impacted the number of detected lipids. Within the mouse brain, DHB coating resulted in the most detected lipids, while DAN coating resulted in the least. When compared to the CHCA spectra from unfixed tissue, 14 lipids were no longer detectable, and there was loss of signal intensity for all remaining peaks, with the unfixed tissue signal consistently maintaining a 1.3-10x higher signal than the post-IF MS spectra. While the number and identity of detected lipids varied as a function of instrument parameters and matrix selection, these data demonstrate that many lipids become significantly more difficult to detect once the tissue has been subjected to the multiple washing and fixation steps required for IF experiments.

To demonstrate how these methods are applicable to biological systems, we showed the heterogeneity of a select subset of astrocytes within the granular layer of the cerebellum (Figure 7).

**FIGURE 6:**
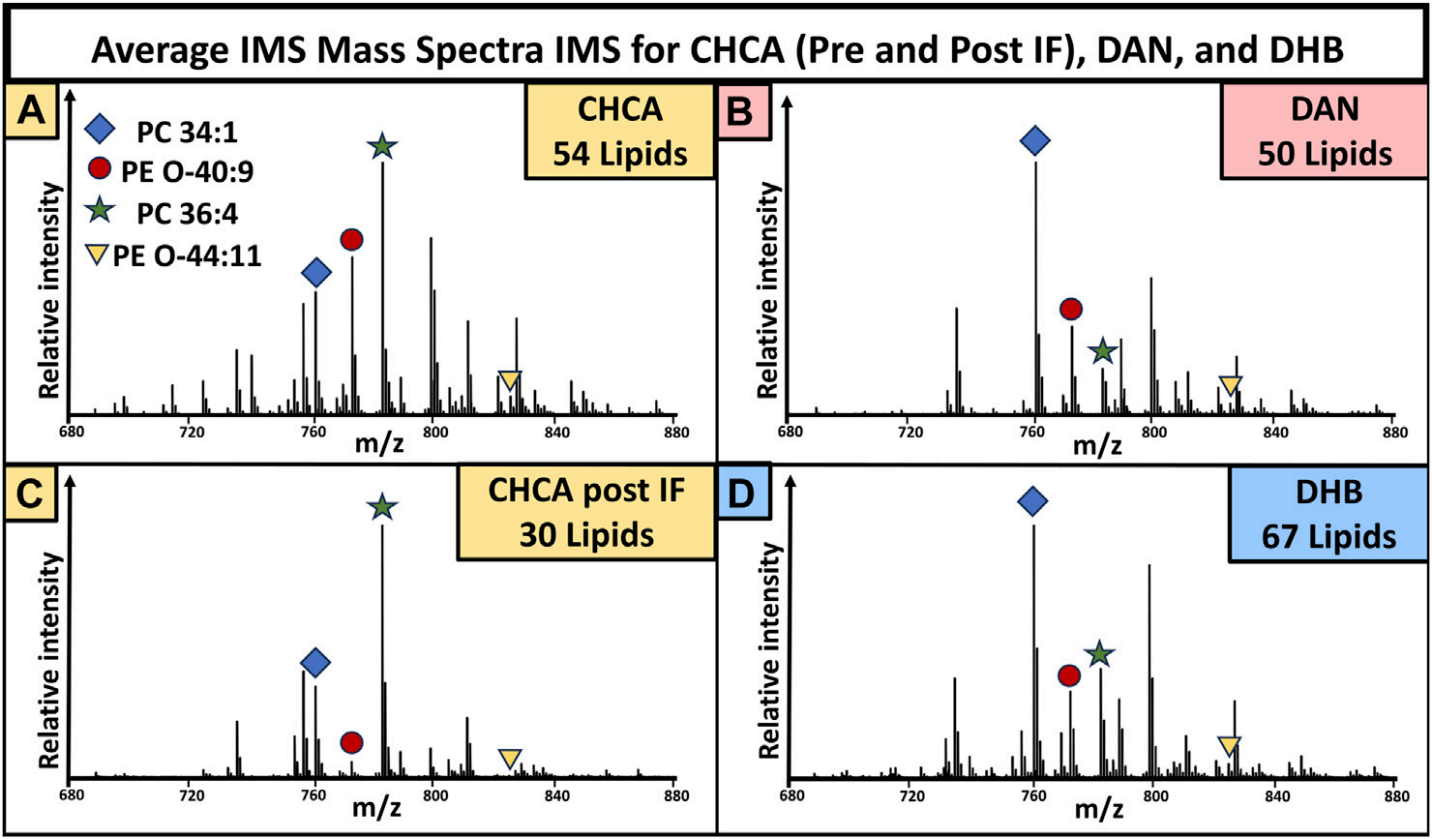
Mass spectra collected from brain tissue used in the varying matrix preparation conditions in Figures 2, 5. Peaks with a signal to noise greater than three were used for lipid totals. The mass spectra for CHCA **(A)**, DAN **(B)**, CHCA post-IF **(C)**, and DHB **(D)** showed 54, 50, 30, and 67 total lipids, respectively. N = 3 for all groups.

**FIGURE 7:**
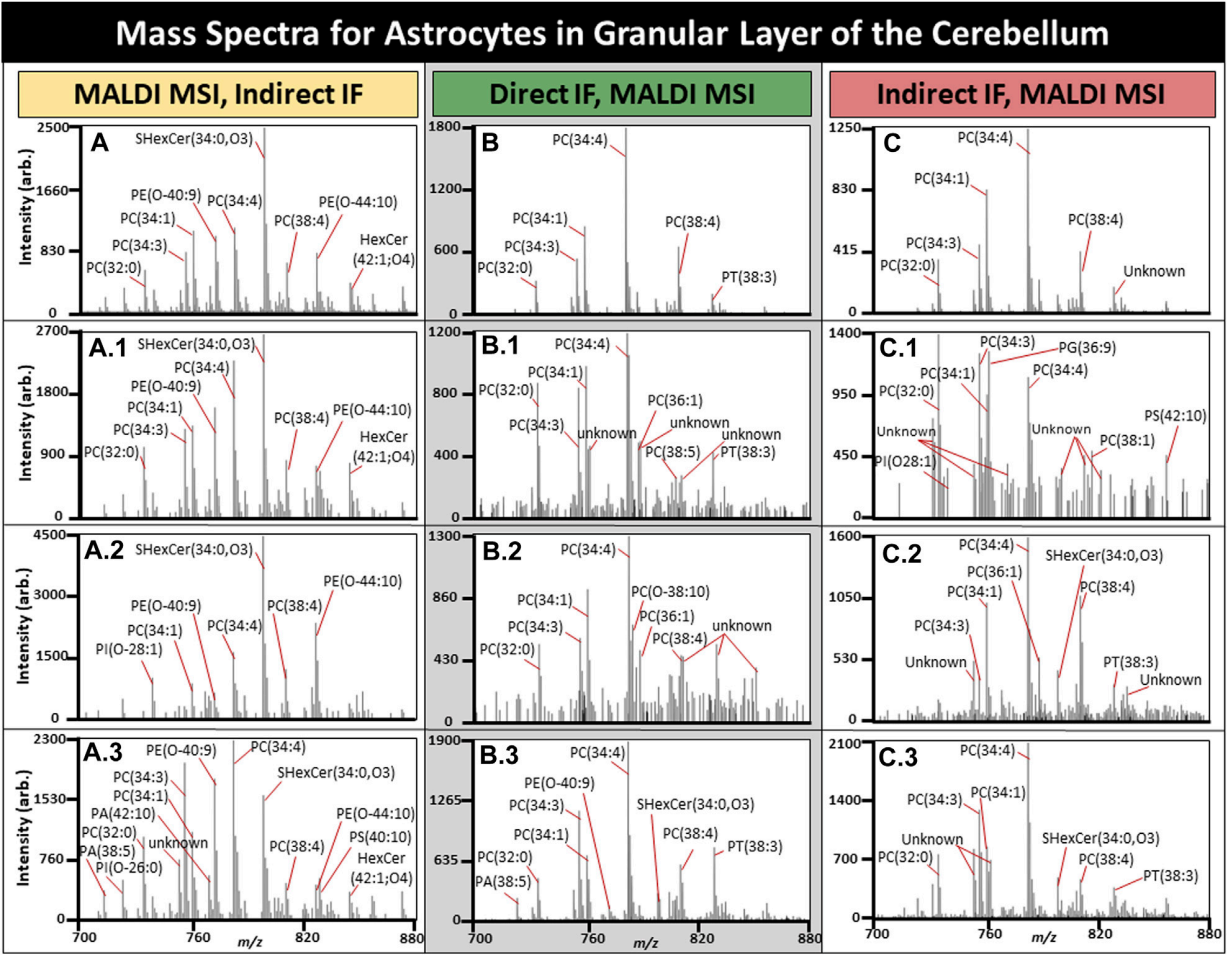
Mass spectra for a subset of astrocytes in the cerebellum. All average spectra contain 150 randomly selected astrocytes from three tissues, 50 astrocytes per tissue. **(A)**. Average mass spectra for 150 astrocytes with three randomly selected astrocyte spectra shown (A1-3) using MALDI MSI performed prior to IF. **(B)**. Average mass spectra for 150 astrocytes when MALDI MSI was performed after direct IF with three randomly selected astrocyte spectra shown (B1-3). **(C)**. Average mass spectra for 150 astrocytes when MALDI MSI was performed after indirect IF with three randomly selected astrocyte spectra shown (C1-3). TIC normalization was performed on these spectra, and n = 3.

Astrocytes have several roles in the brain related to function, with astrocyte dysfunction being observed in a number of neurological diseases including Alzheimer’s disease (AD) (Neumann et al., 2019; Lee et al., 2022). Determining the cell specific metabolomic variation of a diseased state could prove invaluable and lead to improved therapeutics. The average spectrum of 50 randomly selected astrocytes per tissue for MALDI MSI with IF performed afterwards is demonstrated in Figure 7A, MALDI MSI performed after direct in Figure 7B, and indirect IF in Figure 7C. Overall, direct or indirect IF resulted in reduced lipid content if performed prior to MALDI MSI. The base peak for post-IF average spectra was PE (34:4) in addition to the individual astrocytes. Post-IF spectra of individual astrocytes resulted in at least 10 peaks with an undetermined lipid identity and will be the focus of follow-up experiments, although they may be the result of lipids reaction or adducting with the fixative or washing media. SHexCer(34:0,O3) was the base peak for samples where MALDI MSI was performed prior to IF and two of the three randomly selected astrocytes. In every case, the average spectra differed from the individual astrocyte spectra, supporting the need for spatially resolved experiments. In most cases, the individual astrocytes contain lipid peaks that have greatly suppressed signal intensity in the average spectra, such as for the CHCA control where one astrocyte contained PI(O-26:0), PA (38:5), and PA (42:10), all of which were not readily apparent in the average (Fig. A, A.3). Lipid differences in individual cells within the native microenvironment have great applications in the study of disease states such as AD, where activated microglia are a known hallmark of disease but have both beneficial and toxic effects over time that are not fully understood (Hansen et al., 2017).

## Conclusion

Multimodal methods for biological tissue characterization are crucial for the holistic understanding of the molecular and cellular environment that are the foundation for our understanding of health and disease. The type of methods selected will vary depending on the characterization needed in each sample. For the multimodal combination of MALDI MSI and IF, the most reasonable approach that allows for the retention of the most molecular and cellular information was to perform MALDI MSI, first using CHCA as the matrix, followed by IF. This allowed for the best compromise between ion signal diversity and intensity on the one hand and IF image quality on the other. However, CHCA is a matrix used for positive0mode lipid detection. If it were necessary or imperative to detect lipids in negative mode, the use of a different matrix such as DAN may be required, in which case further development of methods and experiments would be required to explore such approaches. In such cases, there may be reasons to further explore if DAN would allow for better MALDI MSI post-IF signal and allow for the retention of the spatial component of the lipids. However, as the inherent nature of IF experimental procedures requires several wash steps with high salt content, the delocalization effects and high signal interference that come with performing IF first remain difficult to overcome.

## Data Availability

The raw data supporting the conclusions of this article will be made available by the authors, without undue reservation.
