# Supplementary material for: Optimized combination of MALDI MSI and immunofluorescence for neuroimaging of lipids within cellular microenvironments
Source: Front Chem. 2024 Feb 9;12:1334209. doi: 10.3389/fchem.2024.1334209 (PMC10884125; doi:10.3389/fchem.2024.1334209)
Supplement: Supplementary file 2 [file DataSheet1.docx]

**Optimized Combination of MALDI MSI and Immunofluorescence for Neuroimaging of Lipids Within Cellular Microenvironments.**

Catelynn C. Shafer^1^, Elizabeth K. Neumann^1,*^

Department of Chemistry, University of California, Davis, Davis, CA 95695

Supplemental Information:


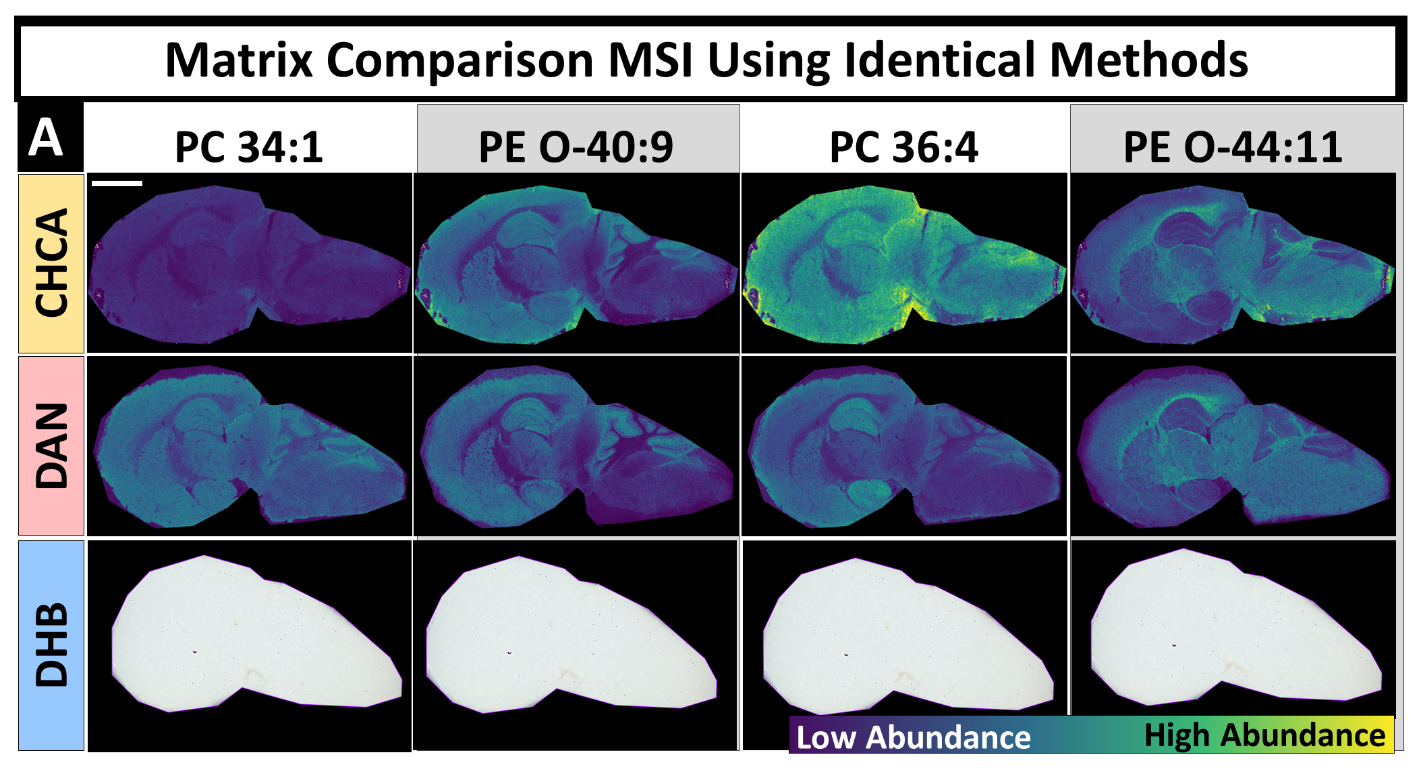


**Figure S1:** resulting MSI images for tissues coated with CHCA, DAN, and DHB using identical methods with 40% laser power.


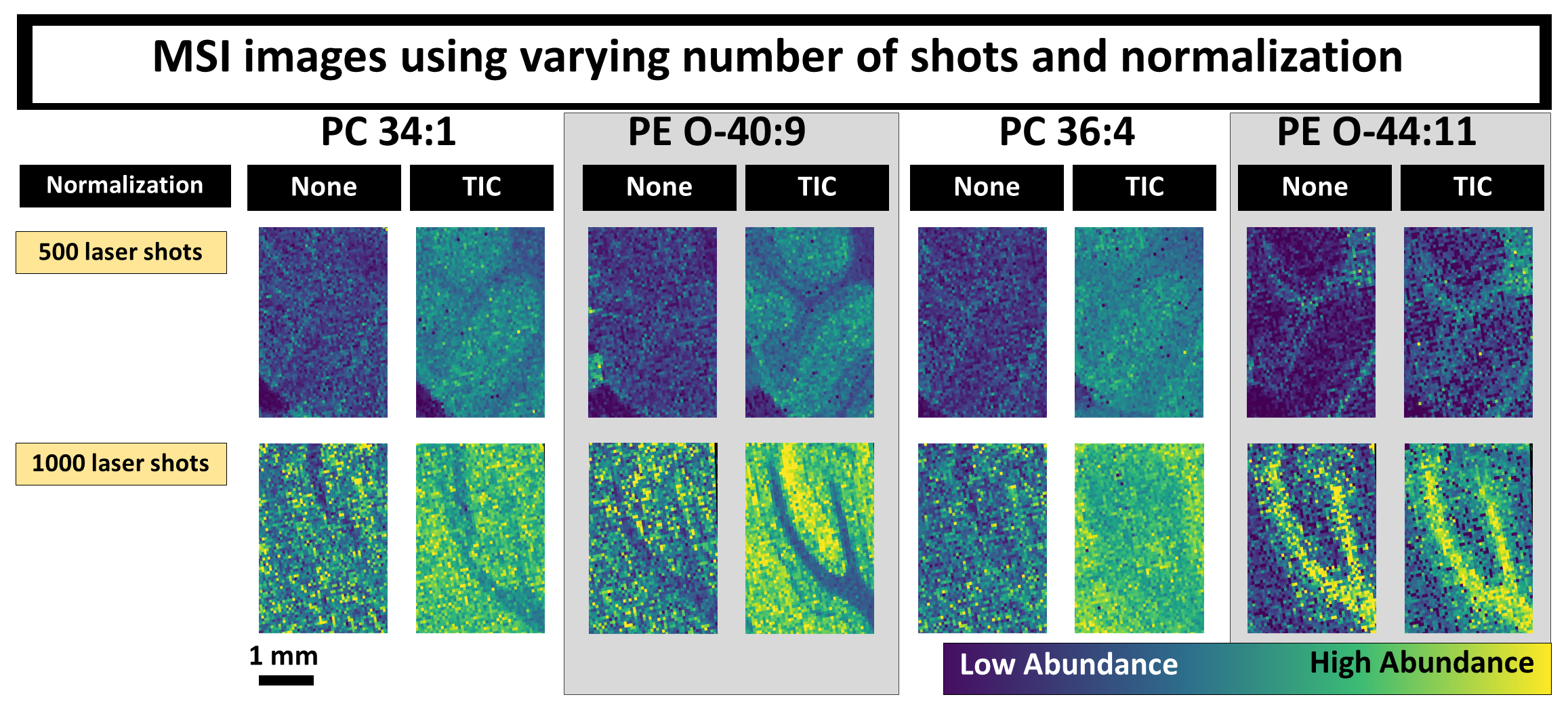


**Figure S2:** MSI images comparing 500 laser shots and 1000 laser shots with no normalization and TIC normalization.
